# Sucrose Is Not the Whole Story: Risk Factors and Oral Health at the Contact (Yakutia, Siberia-16th/19th)

**DOI:** 10.3390/biology10100974

**Published:** 2021-09-27

**Authors:** Eric Crubézy, Sylvie Duchesne, Harilanto Razafindrazaka, Liubomira Romanova, Patrice Gérard, Ameline Alcouffe, Rémi Esclassan, Olga Melnichuk, Innokenty Ushnitsky, Bertrand Ludes, Norbert Telmon, Willy Tegel, Henri Dabernat, Vincent Zvenigorosky, Juan Carlos Prados-Frutos

**Affiliations:** 1 Centre d’Anthropobiologie et de Génomique de Toulouse, Faculté de Médecine Purpan, Université Paul Sabatier Toulouse III, 37 Allées Jules Guesde, 31073 Toulouse, France; sylvie.duchesne@inrap.fr (S.D.); patrice.gerard@univ-tlse3.fr (P.G.); ameline.alcouffe@univ-tlse3.fr (A.A.); remi.esclassan@univ-tlse3.fr (R.E.); norbert.telmon@univ-tlse3.fr (N.T.); henri.dabernat@univ-tlse3.fr (H.D.); 2 Laboratoire International Associé CNRS/Fédération de Russie COSIE, 31073 Toulouse, France; liubomira.romanova@univ-tlse3.fr (L.R.); oa.melnichik@s-vfu.ru (O.M.); bertrand.ludes@u-paris.fr (B.L.); zvenigorosky@unistra.fr (V.Z.); 3 Institut National de Recherches Archéologiques Préventives (INRAP), 13 Rue du Négoce, 31650 Saint-Orens-de-Gameville, France; 4 CNRS, EFS, ADES, Aix Marseille Université, 13005 Marseille, France; razafindrazaka@univ-amu.fr; 5 Institute of Modern Languages and Regional Studies, North-Eastern Federal University, 58 Belinskogo Street, 677000 Yakutsk, Russia; 6 Medical Institute of the North-Eastern Federal University, Ammosov North-Eastern Federal University, 58 Belinskogo Street, 677000 Yakutsk, Russia; incadim@mail.ru; 7 BABEL, CNRS UMR 2029, Université Paris V Descartes, 75006 Paris, France; 8 Faculty of Environment and Natural Resources, Albert-Ludwigs Universität, 79098 Freiburg, Germany; tegel@dendro.de; 9 Department of Medical Specialties and Public Health (Forensic and Legal Medicine Area), Faculty of Health Sciences, Rey Juan Carlos University, Avenida de Atenas s/n, 28933 Madrid, Spain

**Keywords:** wear, tooth loss, abscess, cysts, para-masticatory activities, dendrophagia, tobacco addition, oral exostoses, *torus mandibularis*, *torus palatinus*

## Abstract

**Simple Summary:**

We have studied the dental epidemiology of 96 frozen bodies from north-eastern Siberia (Yakuts) before and after the contact—with an accurate chronology–between Autochthonous and European populations. The peculiarities of the Yakut population are the rarity of cavities and the relative frequency of dental pathologies leading to death. Dental health evolves only two centuries after the contact; assimilation into the Russian Orthodox culture has decreased tooth wear and increased tooth loss. A comparison with historical data suggests that this evolution is not linked to the increasing importance of sucrose, but to the combined action of the substitution of dendrophagia with cereal flour; a decrease in immunity associated with the development of chronic infectious diseases; smoking as well as the mandibular torus: a risk factor favoring apical cysts.

**Abstract:**

(1) Background: contact between indigenous and European populations has often resulted in changes in oral health attributed to the introduction of sucrose. Most studies are per tooth over considerable periods and with few ethnological references. (2) Aim: dental epidemiology of 96 autochthonous frozen bodies from Yakutia between the early 17th century and the late 19th century; comparisons with historical texts and ethnographic data. (3) Material and methods: we use descriptive statistics and discriminant factorial analyses to identify dominant variables in the dataset and compare periods and subjects, considering all variables. (4) Results: the peculiarities of the population are the rarity of cavities and the relative frequency of dental pathologies leading to death. Assimilation into the Russian Orthodox culture has led to decreased tooth wear and an increase in tooth loss. Dental health evolves only two centuries after the contact. (5) Conclusions: the confrontation with historical data suggests that changes are not related to the growing importance of sucrose but to a combined action: the substitution of dendrophagy by cereal flour; the decrease in immunity linked to the development of chronic infectious diseases; tobacco addiction and the mandibular torus: a risk factor promoting apical cysts.

## 1. Introduction

Oral health is the result of an interaction between environmental—natural and cultural—and personal factors, including genetics and life history. Studying the evolution of oral health therefore means considering a multitude of factors relating to cultural and dietary habits and risk factors [1]. In several autochthonous populations [2,3,4,5,6], the transition from a traditional lifestyle to a lifestyle influenced by European colonization resulted in massive epidemiological outbreaks [7] and changes in lifestyle, especially in nutrients consumed [5]. This could have induced a series of modifications in oral pathology, even if important differences in the way in which indigenous subsistence economies were transformed are documented [8]. In the present study, diachronic relationships of oral health were examined from the dentitions of an exceptional sample of 96 frozen bodies or skeletons from Yakutia (oriental Siberia), dated from the early 16th century, a period characterized by a traditional way of life prior to contact with Western populations, to the 19th century, with assimilation in the Russian Orthodox culture [9]. If some results obtained are more or less compatible with those of most studies, the usual interpretation based on the growing importance of sucrose intake cannot be retained, because, in Yakutia, the consumption of sugar is not attested before the 20th century.

The Yakut population is of great interest in studies on the impact of colonization. With the exceptional preservation of the frozen bodies, diachronic studies can be conducted with high precision. In this sample, the impact of colonization can be tracked in 50-year periods (two generations) and the importance of historical and archaeological data can be matched with biological data [10], as well as isotopic analyses [11]. Moreover, this population has been the subject of paleogenetic studies that have demonstrated its homogeneity over time, which makes it possible to affirm that, during this period, the Yakut substratum has not undergone major changes [9,12,13]. In addition, the exceptional conservation of the teeth allows us to work as paleopathologists by studying the frequency of lesions and their evolution over time, but also as contemporary stomatologists by focusing on individual data and comparing the subjects in relation to each other. Thus, the recent history of Yakutia and the societal and cultural changes that followed Russian colonization [10,11] provide a natural experiment on human ecology and anthropobiology in the sense of the relations between archaeological, historical and biological data.

## 2. Materials and Methods

**Figure 1:**
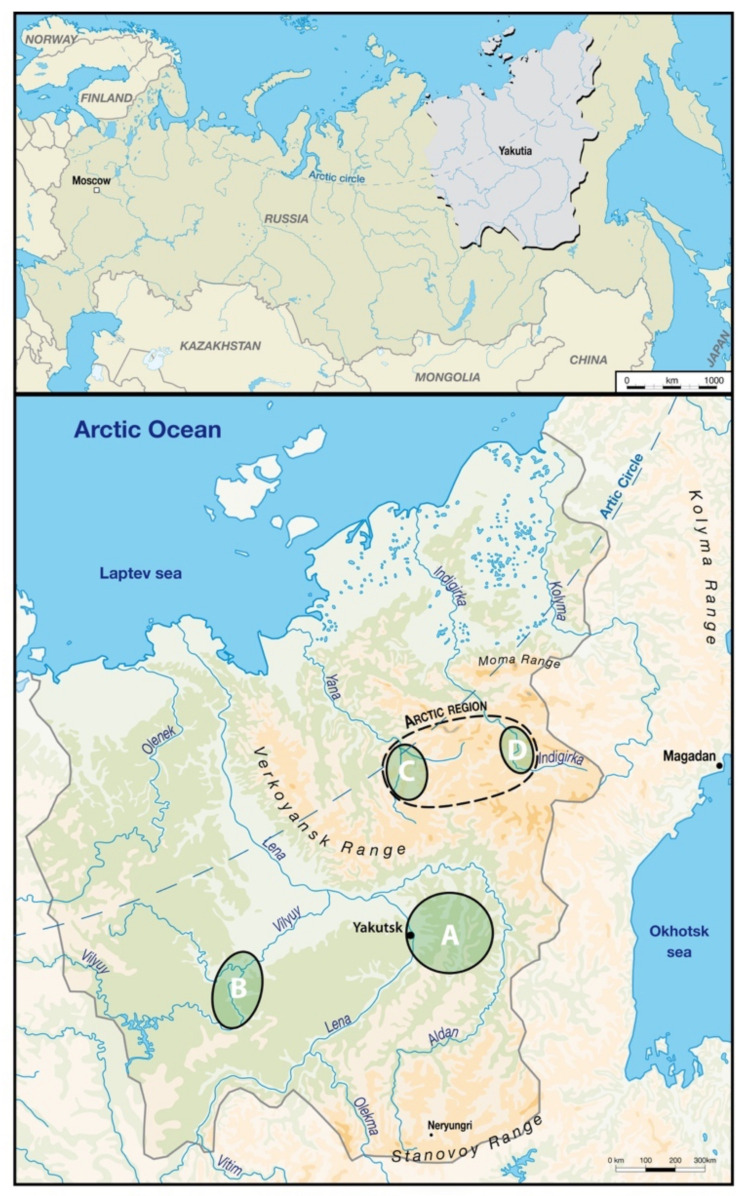
Location of Yakutia and excavation areas (regions). A: Central Yakutia, B: Vilyuy, C: Verkhoyansk, D: Oymyakon. P. Gerard according to © geoatlas.

The Yakuts represent the largest ethnic group of the Sakha Republic of Eastern Siberia (Yakutia), with almost half a million individuals, some of them descended from the Xiongnu of Mongolia [14]. We excavated more than 150 tombs [15], of which around 100 were well preserved by permafrost. The excavations took place in 4 different ecological regions of Yakutia (Figure 1). The human remains were divided into four time periods, culturally distinct and confirmed by radiocarbon and dendrochronological dating. These periods are: before 1700 AD, Russians first settled in the area within the first half of the 17th century [16], however, the Russian influence was not yet evident in the graves; from 1700 to 1750 AD, the Russian colonization had a far reaching impact on the social sphere, with the rise of an economic elite in the early 18th century during the Yakut Golden Age which witnessed a lineage taking power in Yakutia [9]; from 1750 to 1800 AD, after the collapse of the Golden Age, notably following massive epidemiological outbreaks of smallpox [17]; after 1800 AD, with the reduction in seasonal mobility, the settlement in villages and a growing Russian influence on the economy and lifestyle of the population [18].

Samples from Verkhoyansk and Oymyakon are classified in the Arctic area. There are ecological and economic variations between regions and slightly different lifestyles that are reflected in the isotopic analysis of skeletons (Romanova et al., 2019). Central Yakutia is a large cattle breeding zone with numerous “alaas” (thermokarstic lakes surrounded by pastures) (Crubézy and Alexeev, 2013). The Vilyuy River Basin is very humid and is known for its abundance of fish (Maak, 1887). Verkhoyansk and Oymyakon in the Arctic area are regions of north Yakutia rich in wild game where hunting continues to provide a part of the diet of current populations.

### 2.1. Analysis of the Variables

**Figure 2:**
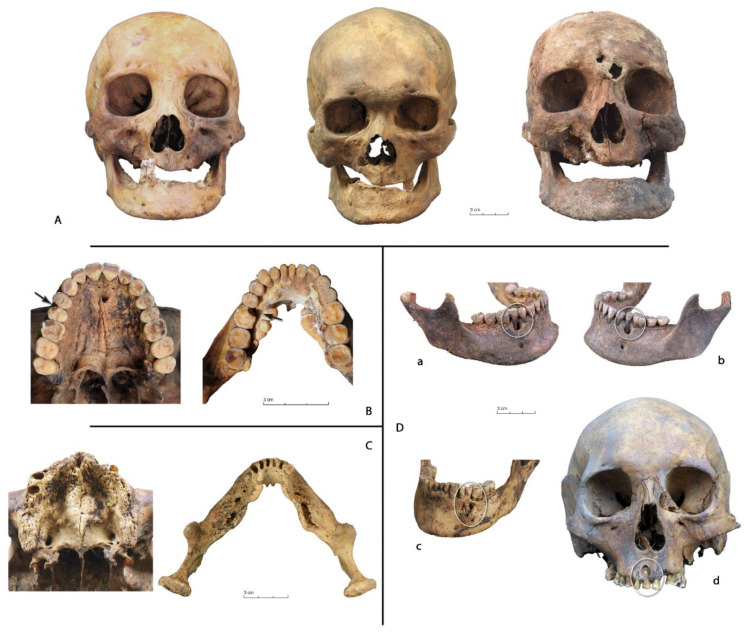
Ante-mortem losses. Front view of subjects with numerous ante-mortem losses, especially in the maxilla (Ken Ebé 8, Female, after 1800 AD, Central Yakutia), in the mandible (Tungus Keul 5, Male, before 1700 AD, Vilyui), or in the two totally toothless subjects (At Daban 7, Male, 1700–1750 AD, Central Yakutia). (**A**) Figure 1. Male, before 1700, Central Yakutia) and on the mandible on the lower left first molar (Ougout Keul 1, Male, 1750–1800, Vilyuy). (**B**) Infections. Major bone remodeling on the maxilla (Sola 2, Male, 1700–1750, Central Yakutia) and on the mandible (At Daban 15, Male, 1750–1800, Central Yakutia). (**C**) Periapical voids. (**D**): (**a**,**b**): On the mandible, on the second right and left premolar (Ebuguey 2, Male, before 1700 AD, Oymyakon); (**c**): With an abscess, left premolar, (Sobolokh, Female, 1700–1750 AD, Oymyakon); (**d**): on the right central incisor (Sobolokh, Female, 1700–1750 AD, Oymyakon). © P. Gérard.

Our corpus includes 96 subjects, divided into 93 adults and 3 adolescents (of which dental germs of the third molars were coded separately). Of the 148 subjects available at the excavation, we excluded: (i) children, because the sample was insufficient and there was a large dispersion by age groups; (ii) 15 adults for whom the data—for various reasons—were not exhaustive [19]. Before 1700 AD, only men were buried. After 1700 AD, both men and women were buried. We cannot guarantee that this is a random draw from the population [9]; differences in status between subjects could be one of the starting hypotheses to explain some differences. Age estimation was performed for adolescents according to dental [20,21,22] and bone growth and maturation processes [23]. For adults, it was conducted according to bone maturation of the innominate bone and clavicle and morphological changes in the sacro-pelvic surface [24]. Subjects were then divided into three classes: under 30 years of age, 30 to 50 years of age and over 50 years of age. Sexual diagnosis was phenotypically established and confirmed by genetic data. For each subject, the dental sphere was examined in the field, during autopsy of bodies, by two senior researchers (SD, EC) who compared their results in a double-blind fashion to end up with a consensual description. Finally, some lesions were photo-examined by an odontostomatologist (RE) to confirm what had been determined during the autopsy. We determined, by macroscopic examination, six indicators of the state of oral health: antemortem tooth loss, dental wear, carious lesions, granulomas and/or cysts, active abscesses and fractures of the crown [25]. Major complications related to active abscesses, infection of adjacent tissue, have also been recorded. Four morphological indicators, dental agenesis, microdontia, oral exostoses including *torus mandibularis* and *torus palatinus*, were also evaluated because of their very high frequency and their unusual development—among the most important ever described—for some in this sample. Antemortem tooth loss was diagnosed when the tooth was missing and bone healing was ongoing or complete (Figure 2A). For the study by individual, we distinguished between those individuals with more or less than 21 teeth. According to the World Health Organization (WHO), adults should have a minimum of 21 functional teeth to provide the ability to experience a good dietary intake [26]. Wear is used to denote the resulting loss of dental hard tissue from any combination of attrition, abrasion and corrosion [27]. There are numerous classifications in the literature [27], especially Molnars’s [28] and others with complex quantitative assessments of wear facets and their angles, which require time and sometimes equipment that were unsuitable for our field studies. Methods using classifications with multiple stages of dental wear provide greater resolution of the degree of dentine exposure. Furthermore, error rates were found within and between observers, even for the frequently used Molnar classification [29]. Intra-observer errors greater than 5% and inter-observer errors greater than 10% were found, a fact confirmed in clinical studies [30]. We therefore used a method in five stages [31] which is easy to use, widely used in medieval populations from continental Europe [32,33] and gives minimal intra- and inter-observer errors [33]. This method perfectly met our objective of assessing whether wear differed between time periods or regions. For future comparisons, our correspondences with Molnar’s classification [29] are as follows: grade 0 = unworn corresponding to Molnar 1; grade 1 = wear facets, no observable dentin corresponding to Molnar 2; grade 2 = small dentin patch corresponds to Molnar 3 and Molnar 4; grade 3 = secondary dentin slight to extensive, corresponding to Molnar 5 and Molnar 6; grade 4 = extensive secondary dentin corresponding to Molnar 7 and Molnar 8.

**Figure 3:**
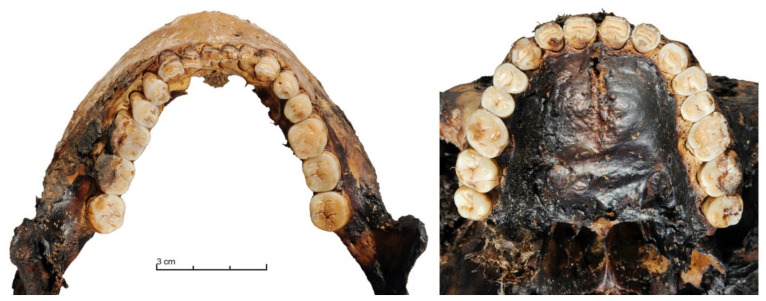
Maxilla and mandible of the subject of Kerdugen in Verkhoyansk, Arctic area. Adult male from the elite, dated from 1700 AD to 1750 AD, with more dental wear on the incisors and canines than on the molars. © P. Gérard.

For the study by individual, we looked for: (i) para-masticatory activities, especially the tanning of hides with a homogeneous dental wear on all the teeth [31]; (ii) a special pattern found in two contemporary elderly women in the Verkhoyansk Arctic area. These two elderly women presented more dental wear on the incisors and canines than on the molars. It was spontaneously declared that the pattern of dental wear of their teeth is the result of bone sucking and that their teeth fell spontaneously without hurting them. For the last aspect of our research, we selected the 40 subjects for whom a maximum of two teeth were missing (Figure 3). Indeed, we observed that when more teeth are missing, the dental wear patterns between the blocks of incisors, canines, premolars and molars are too heterogeneous.

Any cavity affecting enamel, dentin, cement or pulp was counted as a carious lesion [34]. Because some carious lesions may be obscured by dental wear, may manifest microscopically or may only be observable radiographically, our observations represent a minimum estimate of prevalence.

Periapical voids were recognized in their advanced stage (2 to 3 mm). This could be a bias factor, however, even with routine roentgenograms, early stages of bone disease cannot be detected, nor can the size of a rarefied area on the roentgenogram be correlated with the amount of tissue destruction [35]. Periapical voids is a general term for alveolar cavities, as it not does not use condition-specific terminology because some pathologies are often indistinguishable on dry bone [36]. We differentiated between “periapical granulomas and/or radicular cysts” on the one hand (the latter lesions corresponding to the advanced stage of the first, they can only be distinguished in living organisms by histological analysis, therefore diagnoses are not available in skeletal material) Figure 2D(a,b,d), and the “active abscess” Figure 2D(c) on the other hand. Periapical granulomas and radicular cysts have a smooth-walled chamber [37], and active abscesses have interior walls which are not smooth and areas of active bone remodeling around the edges of the sinus [38]. The name “active abscess” covers two infections of different origins. The first is an acute periapical abscess with an abscess formation which occurs rapidly and a periapical destruction of bone—often moderate—with a perforation of the adjacent cortical bone [36]. The second is a chronic periapical infection and/or chronic dental abscess [36] which develops from a periapical granuloma by the accumulation of pus, with fistula formation that perforated the alveolar bone [38]. It should be noted that many radicular cysts remain confined within the normal profile, however, some grow to form a surface bulge in which the outer layer of bone becomes very thin. Dental cysts are usually quite small and may be left unrecognized or be mistaken for a simple abscess. In some cases, there are some active periosteal reactions not only around the edges of the sinus but on a large portion of the maxilla or the mandible, which is a sign of an infection that has spread to the surrounding tissue (cellulite of the floor of the mouth or sinus infection). These bone infections, because of their severity [39], were coded separately (and the abscesses were coded with the active abscesses) (Figure 2C).

Traumatic lesions consisted of fractures of the crown (Figure 2B) occurring when the jaw acts as a nutcracker—the cracked tooth syndrome [40,41].

**Figure 4:**
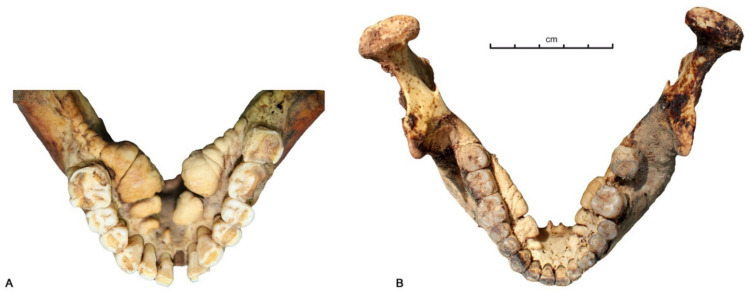
*Tori mandibularis* (TM) of subject Bekh Alaas 1 (**A**) et Eletcheï 3 in Central Yakutia (**B**). © P. Gérard.

Oral exostoses or Tori are a bony protuberance located in the mandibular (*torus mandibularis* TM) (Figure 4), the palatal (*Torus palatinus* TP) and the maxillary bones (*Torus maxillaris* TM). Their prevalence has been extensively studied in many diverse populations. If they present extreme variations in shape and size [42], it is not clear if these are solely an anatomical variation or if they can be linked to other oral pathologies. In this study, *Tori* were categorized by ordinal coding such as continuity, using the Movsesjan scale [43]; the size of the torus is coded by size class of development level [44,45], extent and number of teeth involved [42] and anatomical position (antero-posterior and vertical [42]. As previous studies have described, some discomfort can occur and can even cause pathologies if their dimensions become too large [43]; we have then tested the link between the prevalence of Tori, their size and their anatomical position with some other pathologies such as dental teeth loss and radicular cysts.

### 2.2. Statistical Analyses

Descriptive statistics of discrete qualitative and quantitative variables were computed using the R programming language, as well as all statistical tests. Mann–Whitney tests were applied to independent variables: numbers of teeth, agenesis, microdontia, abscesses, periapical voids, fractures, cavities and five stages of tooth wear (Welch’s *t*-test could only be applied to some levels of tooth wear, although other variables would likely follow normal distributions if more subjects were available). Unless otherwise specified, all the *p*-values presented are for Mann-Whitney tests. The threshold for the significance of *p*-values for tests was set at 0.05. A Factor Analysis of Mixed Data was used to describe the different periods and a Factorial Discriminant Analysis (FDA), similar to logistic regression, was used to identify dominant variables in the dataset and specify the probability of subjects being members of each period [46]. A Wilks’ Lambda test was used to test the quality of the discrimination [47]. The 361 variables per subject were aggregated into 13 summary variables per subject: each subject was represented by his or her total number of teeth, the total number of agenesis, wear/from 0 to 4, microdontia, total number of abscesses, the total number of periapical granulomas and radicular cysts (PGRC), total number of fractures, total number of caries and total number of infections. These variables were used to compare four time periods, four geographical regions, three age classes and two genders.

## 3. Results

A total of 96 subjects were analysed (Table 1), and most of the subjects were found in central Yakutia (64.58%), followed by the Arctic (23.96%) and the Vilyuy River Basin (11.46%). After 1750 AD, the spatial distribution of the subjects was more homogeneous, however, a greater number of subjects were always excavated in central Yakutia. The majority of the subjects in the sample had an estimated age between 30 and 50 years old, followed by subjects over 50 years old and subjects under 30 years old. The distribution of ages by period and by region was heterogeneous, however, the differences by age group according to periods was not significant. In the sample of 96 subjects, 2992 positions (corresponding to the position of the 32 teeth of the maxilla and mandible) could be analysed. A total of 80 teeth could not be evaluated due to loss after exhumation or poor state of conservation. The highest percentage of missing teeth were in positions 28, 27, 24 and 13 (4.17%) (Table 2).

### 3.1. Data Analysis per Tooth (Data Description)

Presence and absence of teeth (Table 3): the teeth most commonly missing were the upper third molars: 18 (46.88%), 28 (53.25%) and, in the anterior sector, incisor 11 (40.63%). Of the subjects, 37.7% (36/96) had fewer than 21 teeth, this affected subjects over 50 (53% of this age group), then those between 30 and 50 (26% of this age group) and, finally, those under 30 (5.5% of this age group). Older subjects thus had significantly fewer teeth than younger ones (Mann–Whitney test *p*-values all inferior to 0.05 between age groups). There were no differences between genders (*p*-value 0.2409) and between regions. However, the subjects living after 1800 AD had significantly fewer teeth than subjects from all other periods (Mann–Whitney test *p*-values all inferior to 0.05).

**Figure 5:**
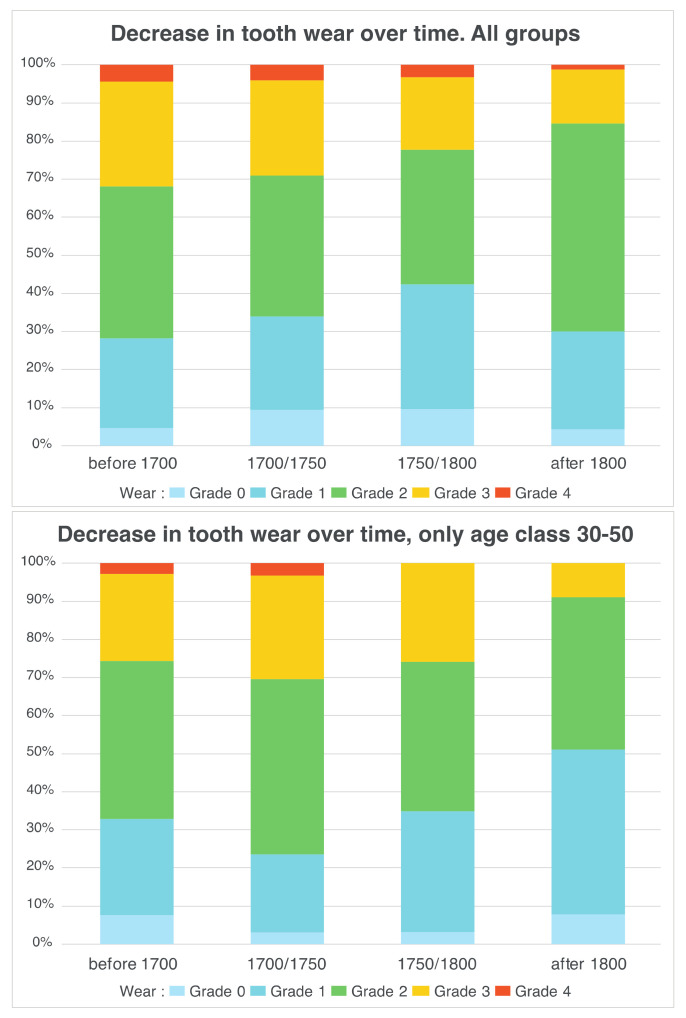
Decrease in tooth wear over time. All groups (**top**). Decrease in tooth wear over time, age class 30–50 (**bottom**).

Tooth wear: the highest percentage of teeth with no dental wear were the third erupted molars: 48 (25.0%), 38 (22.9%), 18 (16.7%) and 28 (11.5%). The teeth with the most grade 1 dental wear were the premolars: 15 (32.3%), followed by 25, 37, 44 and 45 (28.1%); for grade 2 dental wear, incisors and canines: 32 (37.5%), 43 (36.5%), 34, 33 and 42 (35.4%); for grade 3 dental wear, incisors and canines: 32 and 42 (29.2%), followed by 33 and 43 (27.1%, respectively); finally, for grade of dental wear 4: 14 (7.3%) and 23 (5.2%) (Table 4). Tooth wear increased with age. This is demonstrated by the comparison within each damage grade. The number of teeth worn at levels 0 and 1 is more important in younger subjects than in middle-aged subjects and, in turn, than in older subjects (all Mann–Whitney test *p*-values inferior to 0.05). For grade 2 of wear, young and middle-aged subjects are similar, however, older subjects possessed significantly fewer teeth worn at grade 2 (*p*-values inferior to 0.05). Teeth worn at grades 3 and 4 are more common in middle-aged and older subjects than in young subjects (*p*-values inferior to 0.05 for both Mann–Whitney and/or Welch’s *t*-tests). The two older age classes, however, showed similar numbers of teeth worn at grades 3 and 4. Men showed lower levels of wear than women, however, tests show that these differences are not significant; *p*-values for all wear grades were superior to 0.05, only the tests for grades 1 (Welch’s *t*-test) and 2 (Mann–Whitney test) showed *p*-values inferior to 0.1. We also show differences between periods (Figure 5): there is a significant drop (Mann–Whitney test p-values inferior to 0.05) in grade 3 wear between the first two periods (before 1700 AD and 1700/1750 AD) and the last period (after 1800) and a significant increase in the prevalence of grade 1 wear in the last period (although tests between the earliest and the most recent period show a *p*-value of 0.08, other *p*-values being inferior to 0.05).

Fractures and cavities: we did not find a large percentage of fractures of the crown, but in order of frequency, 16 (4.2%), 36 (3.1%), 26 and 15 (2.1%) were fractured and the apparent differences between males and females were not significant (Mann–Whitney test, *p*-value = 0.09625). Cavities were scarce. They were only found in 36 (2%) and 37 (2% and 1%) of subjects. There was no difference was found between age groups, periods, regions and sexes.

Agenesis was more frequent in third molars: 18 and 28 (18 and 14%), 48 (13%) and 38 (8%), followed by incisors: 12 (1%), 22 (1%) and 42 (1%), some premolars: 34 (1%) or molars: 17 (1%). There was no difference between age groups, periods or sexes. There was more agenesis in the Indigirka than in the Vilyuy.

“Periapical granulomas and radicular cysts” were more frequent in the first molars: 36 (8.5%), 26 (7.4%), 46 (6.3%) and 16 (6.3%).

“Active abscesses” were found in the posterior sector: 26 (4%), 27 and 28 (3%), 38, 18, 16, and 17 (2%). Five subjects (5.1% of the dataset), four males and one female (one anterior to 1700 AD, two between 1700–1750 AD and two between 1750–1800 AD), had an active abscess with active periosteal reactions at the time of death on a large portion of the maxillary or the mandible.

Of the 78 subjects for which *Tori* was studied, 55 (31% of female and 69% of male) had *torus mandibularis* (TM) and 10 had a *torus maxillaris*. There is a link between the presence of TM and radicular cysts (p 0.0242 One-tailed Mann–Whitney test) but no direct link with the dental loss. There is no statistical difference between: sex and periods; the extent of TM with the number of concerned teeth, the development level of the TM with dental loss and the radicular cysts; the presence of cysts and the vertical position of the TM [45]; the antero-posterior anatomical position of TM and the location of the cysts.

### 3.2. Between-Subject Comparisons for Periods and Variables

**Figure 6:**
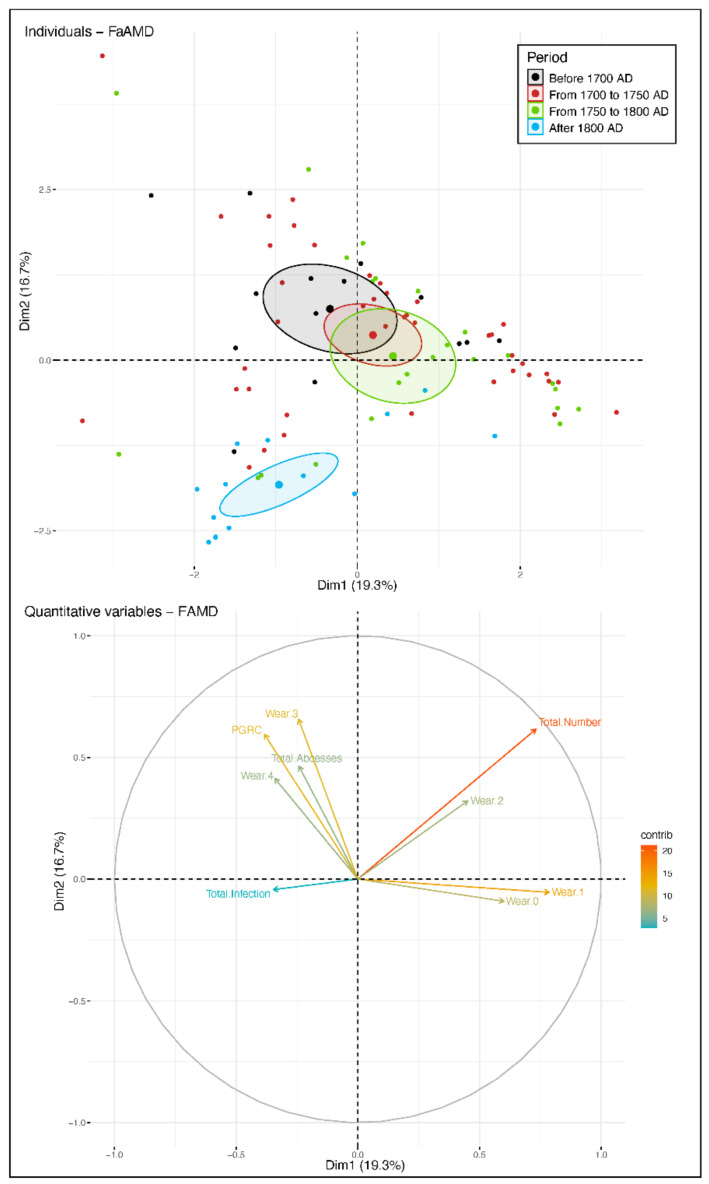
Factorial analyses. Each subject is represented by his or her total number of teeth, wear/from 0 to 4, total number of abscesses, the total number of periapical granulomas and radicular cysts (PGRC), total number of infections. Superior. Factor Analysis of Mixed Data, first factorial plane. Concentration ellipses at 95% threshold centred on the centre of gravity of the four periods. If the ellipses intersect, the groups are not significantly different. Inferior Correlations circle of variables.

Factor Analysis of Mixed Data (Figure 6): on the first factorial plane (36% of the variability), only the last period (post-1800 AD) is different from the others, mainly due to tooth loss and, to a lesser extent, to infections. FDA: agenesis, microdontia, fractures and caries are underrepresented in all periods and, finally, we only retained the total number of teeth, wear/from 0 to 4, the total number of abscesses, the total number of periapical granulomas and radicular cysts (PGRC) and the total number of infections. The quality of the FDA discrimination is not significant (Wilks’ Lambda test, *p*-value: 0.18). Only the barycentre of the post-1800 AD period deviates from the other three, and the discrimination is essentially related to tooth loss. Of the subjects from 1700–1750 AD, 74% are correctly attributed, 47% for those after 1800 AD and only 36% and 20%, respectively, from 1750/1800 and ante 1700 AD.

Five subjects of both genders, all over 50 years (around 15% of this age group), were entirely edentulous. Ten subjects of both genders (five are over 50 years old) were entirely edentulous only in the maxilla. Of these 10, 4 still had between 2 and 6 teeth and one had 12 teeth on the mandible. No subject in the entire sample was only edentulous in the mandible. Adult subjects with only two or three teeth were posterior to 1700 AD. We observed no differences in teeth number between the regions (all *p*-values superior to 0.05), however, there are significant differences between age groups, with more teeth in young people than in old people (Mann–Whitney test *p*-values inferior to 0.05) and between periods, with fewer teeth post 1800 AD than in all three previous periods: ante 1700 AD (*p* = 0.0133), 1700/1750 AD (*p* = 0.0052) and 1750/1800 AD (*p* = 0.0114). Between periods anterior to 1800 AD, there was no significant difference (all *p*-values above 0.05).

We identified patterns similar to the two elderly women in Verkhoyansk, with more dental wear on the incisors (inferior and superior) than on the molars, in 47% of subjects (19/40) who had less than two missing teeth (Figure 3). In the same sample, 20% (8/40) had homogeneous dental wear on all their teeth. For the others, we found right and left asymmetries in dental wear and/or differences between the maxilla and mandible. Interestingly, for three subjects, the maxillary incisors showed more wear than the other teeth, while, on the mandible, homogeneous dental wear was observed on all the teeth, however, less marked than for the mandibular anterior teeth. There were no significant differences between regions, sexes or periods.

## 4. Discussion

It is only after 1800 AD, when Yakutia becomes a Russian Orthodox society [10], about 200 years after the first contact, that we can detect a difference in oral health in the form of a decrease in the total number of teeth and wear. However, the reduction in wear and the increase in dental loss are noticeable as early as 1700 AD, i.e., a century earlier. In fact, over the four periods, variability remains high, as shown by the low percentage of subjects correctly attributed to a period by the AFD. Bias factors such as differences in age at death and gender may be involved, however, historical data (see below) also suggest variability between subjects since, in the 19th century, part of the population was still using wood-based flour. After a golden age, between 1700 and 1750 AD [9,10], and a relatively homogeneous sample, the population may have entered a transition period (1750/1800 AD) which continues very late, perhaps until the early 20th century. However, over time, some specificities of the samples are: (i) the rarity of dental cavities compared to other populations at the contact [2,48]; (ii) the frequency of *torus mandibularis* and their link with radicular cysts; (iii) the frequency (more than 5%) of dental pathologies at the origin of death; active abscess with active periosteal reactions at the time of death.

Wear decrease is one of the most important features of our data set, especially for the highest wear levels (grade 3 and 4) in more recent periods regardless of age groups. We described two special patterns: (i) a homogeneous dental wear on all the teeth, classically related to the tanning of hides [31]; (ii) a more special one related to the bone sucking habit. Yakuts were and still are large consumers of horse meat, however, isotopic studies have demonstrated that they consumed more meat before than after 1700 AD and that, in Verkhoyansk, Arctic area, the diet has always been more based on meat consumption than elsewhere [11]. We cannot only hypothesize the decrease in meat consumption and the habit of bone sucking. Reduction in tooth wear is related to food that, apart from meat, should be less abrasive. During the period under consideration, there was also a significant decrease in the consumption of wood bark. Vegetable food, especially wood, had an important role in the diet of the Yakuts and the wood-based flour was obviously rougher than cereal flour. A household in Central Yakutia consumed 640 kg of plants, 70 kg of roots, 50 to 60 kg of sapwood and 80 kg of berries per year [49,50]. In Verkhoyansk, between 30 and 50 kg of roots were prepared, and the population of Oymyakon consumed up to 480 kg of plants on average [49,50]. The phloem of pines (*Pinus silvestris* L.)—in Central and South Yakutia—was more appreciated than that of larches (*Larix gmelinii* or *Larix dahurica turcz*) in the far north, and this “wood flour” was used to make porridge or, more rarely, a cake. A poor family consumed up to 256 kg of sapwood flour per year [51] and, in 1737 AD, Khariton Laptev notes that the usual food of the Yakuts was sapwood of the tree with milk [52]. This dendrophagy was also known among Alaskan Tlingits, Finns, Samoyeds and Sami [50,53,54]. The disappearance of dendropagy is linked to the development of ploughing and agriculture in the 19th century [55], but it will persist in northern regions and, at the end of the 19th century, only 10% of the population had access to flour [56]. This disappearance will moreover be progressive: in 1838 AD, there were about 50 bakeries in Yakutsk and more than 800 in 1860 AD [57]; however, as Middendorf [51] pointed out, only rich people could add flour to their dish.

Throughout the period studied, the rate of cavities was very low and slightly different from that of non-agriculturalist indigenous west Siberian people of the same period [58] or even pre-contact Inuit [59]. Sucrose plays a dominant part in the aetiology of cavities and other sugars are, to a lesser extent, also incriminated. Caries are uncommon in people living on a diet high in starch and low in refined sugar; however, they become more common when refined sugars, usually sucrose, are introduced into their diet [60]. The level of sugar consumption under which most of the population will not get dental caries is 15 kg/person/year [61] and the Yakuts never hit that rate. Sugar was sold on the market of Yakutsk in the late eighteenth century, and the volume of sugar traded increased in the 19th century [62], probably due to the beginning of sugar production in 1809 AD in Russia from domestic raw materials such as white beet. However, the sugar mentioned in the texts was either sold to the Russians or consumed on minimal quantities by the Yakuts until the end of the 20th century. It is only in the 21st century, while the consumption of sucrose has increased considerably in Siberia and Yakutia, that the incidence of caries has increased dramatically [63]. This rarity of cavities certainly explains the rarity of certain phenotypes such as edentulism, since only 15% of subjects over 50 years old were completely edentulous, a frequency similar to the lower percentage found in many European populations from the 20th century, such as Swedish women in 1969 [64], while this incidence was 30 to 60% in other countries among 65-year-old subjects [8,65]. We can note that these edentulism problems have led to severe maxillary atrophy, the collapse of the large defects and significant volume loss, decreased bone quality, expansions of the sinus floor in the maxillary molar region and, finally, aesthetic problems. All these lesions, still well known today [66,67,68], currently require specific treatments [69,70].

**Figure 7:**
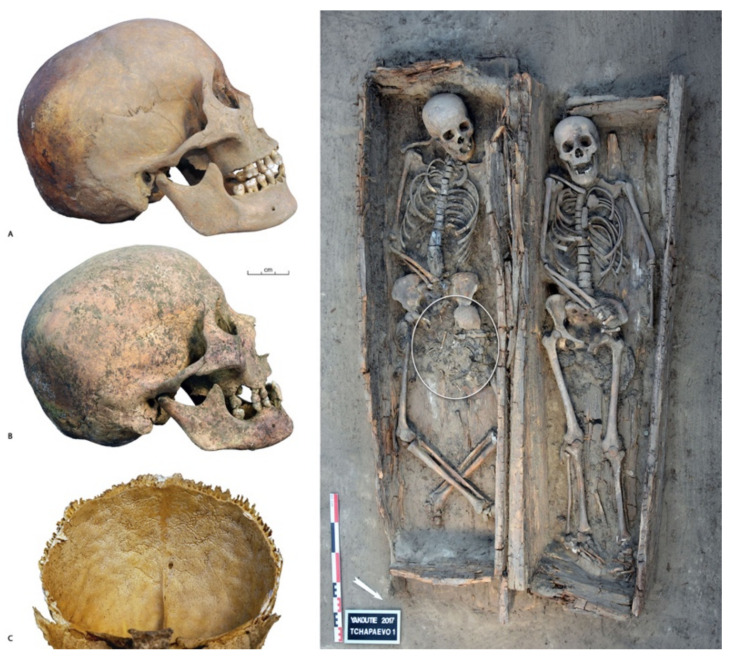
Tchapaevo grave 1. Central Yakutia, early 19th century. Triple grave made up of two joined coffins, placed at the same time, each containing a young woman (20 to 25 years old). The older one (skull **B**) had newborn baby between her thighs (circled in white). The newborn baby, born at term, died around birth; the woman buried with him presents (skull **B**) an infection of the base of the skull, with a voluminous periosteal reaction at the internal part of the carotid canal and a fine periosteal reaction of the occipital body as well as periosteal appositions on the internal face of the frontal (**C**) evoking meningitis. She has agenesis of the right upper third molar and, despite her young age, ante-mortem loss of the upper incisors and the left upper first molar, post-mortem loss of the canines and the left first premolar. The left upper molar has a fistulized abscess with bone loss. The mandible shows agenesis of the last right molar, ante-mortem loss of the first molars, right canine, central incisors and post-mortem loss of the second left molar. The young woman buried next to her (skull **A**) has the germs of her third molars, an ante-mortem loss of the left upper first and second molars (post-mortem loss of the others teeth). © P. Gérard.

The increase in dental loss over time must be explained in the absence of cavities and decline in dental wear over time that compromise the integrity of the pulp chamber. When teeth have been lost and the alveolar process has remodeled them, it is often difficult to reconstruct the pathological process involved [34]; however, periapical granulomas and radicular cysts were numerous in the samples studied, and they could easily lead to the expulsion of the tooth. When we cross the evolution of their frequency and that of ante-mortem tooth losses over time, increased tooth loss was related to a decrease in cysts. This is explained by the fact that the lost teeth can no longer have a radicular cyst or become infected. A great number of the variables measured are associated with oral health, and there is no consensus as to whether dental disease-related or socio-behavioural factors are the most important risk indicators for tooth loss [8,71,72]. In the present case, several processes, certainly interconnected, can be evoked to explore the increase in dental loss associated with contact with Europeans and, more specifically, with the integration of the Yakuts into Russian Orthodox society. Others may have existed, but we have no elements to evoke them, such as changes in biofilms formations [73], plaque-induced gingivitis [74] and gene expression, which benefit today from stem cells implantation [75,76]. These three processes concern a general process of diminished immunity which can be evoked in the face of certain chronic infectious pathologies: (1) an addiction to tobacco in the 19th century associated with an increase in the consumption of black tea and no longer green tea, which was drunk in the 18th century [77]. While some people smoked in the 18th century, in the 19th century, tobacco became widespread [77] and a large part of the population, even older children, became addicted to it [78]. There is a strong, dose-dependent association between tobacco smoking and risk of tooth loss [79] and, in present day populations, with a significantly greater number of implant failures [80]. Exposure to smoke has been associated with caries in several studies, with mechanisms relating to alterations in saliva [81] and the number of missing teeth being higher among smokers of pipes [82], which was the case among the Yakuts [77]. Moreover, in the 19th century, the consumption of green tea, associated in some studies with a decrease in tooth loss [83], disappeared in favor of black tea [77]. There is no evidence suggesting that the Yakuts put sugar in the teas, always consumed today in the countryside, very diluted and without sugar. (2) A risk factor represented by *torus mandibularis*; in our sample, the frequency of *torus mandibularis* is similar from one period to another, however, the presence of a torus is a risk factor for radicular cysts and, therefore, for tooth loss, which could have been fully expressed when living conditions changed. (3) Increased susceptibility to infection, and deficiencies in the immune response, can be considered as the most significant causes of antemortem tooth loss [84]. Susceptibility is generally greater in very young and very old individuals [85], who usually exhibit weaker immune responses. While it is difficult to assess the immune response of subjects, it should be noted that it is only in the 19th century that cases of young adults (20–30 years old) with significant dental loss, sometimes associated with signs of chronic meningitis, were encountered (Figure 7), while most infectious diseases recorded in the 18th century were rapidly fatal, such as smallpox. The population most similar to the Yakut is the population of Eyging Gol, a population of horse breeders in northern Mongolia at the beginning of our era [86] who had a way of life comparable to the Yakuts. In this population, a similar “horse breeders’ pattern” was found, with a rarity of cavities (about 15% of the data set) associated with abscesses and active periosteal reactions reflecting progressive infectious osteitis at the time of death. In this necropolis, as in Yakutia during the 18th and 19th century, infectious diseases were frequent [87]. The question of a decrease in population immunity or the appearance of new germs may be raised at a time when bone tuberculosis was devastating the Yakut population [10,87]. Finally, the decrease in wear and the increase in tooth loss are concomitant in our sample with contact with Europeans. We were able to highlight them during the acculturation after 1800 AD. The non-significance of many statistical tests for other pathologies may be related to the small number of the samples and their selection. Before 1800 AD, we have demonstrated that the Yakuts buried only a part of the population [9]. We can evoke multiple factors to explain the decrease in wear; among them, the reduction of dendrophagy and meat during meals are the best documented. Explaining the increase in losses requires more hypotheses. Only future studies that take into account the infectious epidemiology for all subjects and the determination of the microbiome will allow us to refine these results.

## 5. Conclusions

In Yakutia, concomitant with contact with Europeans, oral health has evolved very gradually. Specific characteristics of this population have remained the same over time, while the increase in tooth loss and the decrease in wear are the most striking. Although we can formulate some hypotheses concerning the evolution of the diet, we can rule out the assumption of an increase in sucrose.

## Figures and Tables

**Table 1:** Distribution of subjects by period, region and age category.

Regions	Central Yakutia			Arctic (Verkhoyansk and Oymyakon)			Vilyuy			Total		
Periods	Below 30	30–50	Above 50	Below 30	30–50	Above 50	Below 30	30–50	Above 50	Below 30	30–50	Above 50
Before 1700	0	8	3	0	0	0	0	0	3	0	8	6
1700–1750	9	13	8	0	6	2	2	1	1	11	20	11
1750–1800	2	9	3	4	2	2	0	0	3	6	11	8
After 1800	0	3	5	1	3	2	0	0	1	1	6	8
Total	11	33	19	5	11	6	2	1	8	18	45	33

**Table 2:** Number of missing teeth and percentage of tooth positions that can be analysed for the totality of the subjects for each of the 32 teeth. N: Number per tooth position; %: percentage per tooth.

Tooth	Missing Teeth		Tooth Positions That Can Be Analysed	
	N	%	N	%
18	0	0.0	96	100.0
17	1	1.0	95	99.0
16	2	2.1	94	98.0
15	2	2.1	94	98.0
14	2	2.1	94	98.0
13	4	4.2	92	96.0
12	3	3.1	93	96.9
11	3	3.1	93	96.9
21	3	3.1	93	96.9
22	2	2.1	94	97.9
23	3	3.1	93	96.9
24	4	4.2	92	95.8
25	3	3.1	93	96.9
26	2	2.1	94	97.9
27	4	4.2	92	95.8
28	4	4.2	92	95.8
38	2	2.1	94	97.9
37	3	3.1	93	96.9
36	3	3.1	93	96.9
35	3	3.1	93	96.9
34	3	3.1	93	96.9
33	3	3.1	93	96.9
32	3	3.1	93	96.9
31	3	3.1	93	96.9
41	2	2.1	94	97.9
42	2	2.1	94	97.9
43	1	1.0	95	99.0
44	2	2.1	94	97.9
45	2	2.1	94	97.9
46	2	2.1	94	97.9
47	2	2.1	94	97.9
48	2	2.1	94	97.9
Total	80		2992	

**Table 3:** Distribution of the number of ante-mortem teeth lost (loss a.m), granulomas and cysts (gran and cyst), fractures of the crown (fracture), caries, agenesis and active abscesses (active.absc.) for each tooth in all subjects. N: number per tooth position; %: percentage per tooth.

Tooth	Loss a.m		Gran and Cyst		Fracture		Caries		Agenesis		Active.absc.	
	N	%	N	%	N	%	N	%	N	%	N	%
18	45	46.9	3	3.1	0	0.0	0	0.0	14	14.6	2	2.1
17	36	37.5	5	5.2	1	1.0	0	0.0	1	1.0	2	2.1
16	31	32.9	6	6.2	4	4.2	0	0.0	0	0.0	2	2.1
15	27	28.1	2	2.1	2	2.1	0	0.0	0	0.0	1	1.0
14	27	28.1	3	3.1	1	1.0	0	0.0	0	0.0	0	0.0
13	29	30.2	2	2.1	0	0.0	0	0.0	0	0.0	1	1.0
12	32	33.3	4	4.2	0	0.0	0	0.0	1	1.0	1	1.0
11	39	40.6	1	1.0	0	0.0	0	0.0	0	0.0	1	1.0
21	36	37.5	1	1.0	0	0.0	0	0.0	0	0.0	1	1.0
22	36	37.5	5	5.2	0	0.0	0	0.0	1	1.0	1	1.0
23	28	29.2	1	1.0	0	0.0	0	0.0	0	0.0	1	1.0
24	28	29.2	0	0.0	0	0.0	0	0.0	0	0.0	1	1.0
25	25	26.0	0	0.0	1	1.0	0	0.0	0	0.0	1	1.0
26	36	37.5	7	7.3	2	2.1	0	0.0	0	0.0	4	4.2
27	35	36.5	4	4.2	1	1.0	0	0.0	0	0.0	3	3.1
28	41	42.7	4	4.2	0	0.0	0	0.0	14	14.6	3	3.1
38	28	29.2	3	3.1	0	0.0	0	0.0	8	8.3	2	2.1
37	33	34.4	3	3.1	1	1.0	1	1.0	0	0.0	1	1.0
36	27	28.1	8	8.3	3	3.1	1	1.0	0	0.0	1	1.0
35	21	21.9	3	3.1	0	0.0	0	0.0	0	0.0	1	1.0
34	18	18.7	2	2.1	0	0.0	0	0.0	1	1.0	1	1.0
33	17	17.7	0	0.0	1	1.0	0	0.0	0	0.0	0	0.0
32	20	20.8	0	0.0	0	0.0	0	0.0	0	0.0	0	0.0
31	27	28.1	0	0.0	0	0.0	0	0.0	0	0.0	0	0.0
41	27	28.1	0	0.0	0	0.0	0	0.0	0	0.0	0	0.0
42	21	21.9	0	0.0	0	0.0	0	0.0	1	1.0	0	0.0
43	17	17.7	2	2.1	0	0.0	0	0.0	0	0.0	0	0.0
44	19	19.8	0	0.0	1	1.0	0	0.0	0	0.0	1	1.0
45	18	18.7	1	1.0	1	1.0	0	0.0	0	0.0	1	1.0
46	25	26.0	6	6.2	1	1.0	0	0.0	0	0.0	1	1.0
47	30	31.2	2	2.1	1	1.0	0	0.0	0	0.0	1	1.0
48	29	30.2	2	2.1	0	0.0	0	0.0	13	13.5	1	1.0
Total	908		80		21		2		54		36	

**Table 4:** Distribution of the number and frequency of the wear depending on the grade (0, 1, 2, 3 or 4). N: Number per tooth position; %: percentage per tooth.

Tooth	Grade 0		Grade 1		Grade 2		Grade 3		Grade 4	
	N	%	N	%	N	%	N	%	N	%
18	16	16.7	9	9.4	7	7.3	2	2.9	0	0.0
17	8	8.3	26	27.1	19	19.8	3	3.1	0	0.0
16	1	1.0	18	18.7	31	32.3	10	10.4	3	3.1
15	4	4.2	31	32.3	19	19.8	10	10.4	3	3.1
14	4	4.2	24	25.0	18	18.7	13	13.5	7	7.3
13	2	2.1	13	13.6	26	27.1	18	18.7	4	4.2
12	0	0.0	6	6.2	25	26.0	23	24.0	4	4.2
11	1	1.0	4	4.2	21	21.9	24	25.0	4	4.2
21	1	1.0	4	4.2	23	24.0	25	26.0	3	3.1
22	0	0.0	5	5.2	25	26.0	24	25.0	3	3.1
23	2	2.1	9	9.4	29	30.2	19	19.8	5	5.2
24	2	2.1	26	27.1	15	15.6	16	16.7	3	3.1
25	4	4.2	27	28.1	20	20.8	14	14.6	2	2.1
26	1	1.0	16	16.7	29	30.2	8	8.3	4	4.2
27	7	7.3	24	25.0	17	17.7	7	7.3	0	0.0
28	11	11.5	11	11.5	6	6.2	4	4.2	0	0.0
38	22	22.9	22	22.9	10	10.4	2	2.1	1	1.0
37	8	8.3	27	28.1	23	24.0	1	1.0	1	1.0
36	0	0.0	18	18.7	31	32.3	14	14.6	3	3.1
35	6	6.2	20	20.8	33	34.4	12	12.5	0	0.0
34	3	3.1	21	21.9	34	35.4	14	14.6	1	1.0
33	2	2.1	11	11.4	34	35.4	26	27.1	2	2.1
32	1	1.0	6	6.2	36	37.5	28	29.2	1	1.0
31	0	0.0	5	5.2	32	33.3	24	25.0	3	3.1
41	0	0.0	6	6.2	30	31.2	22	22.9	4	4.2
42	0	0.0	8	8.3	34	35.4	28	29.2	2	2.1
43	2	2.1	12	12.5	35	36.5	26	27.1	2	2.1
44	3	3.1	27	28.1	30	31.2	11	11.5	3	3.1
45	7	7.3	27	28.1	30	31.2	10	10.4	1	1.0
46	1	1.0	22	22.9	29	30.2	13	13.5	4	4.2
47	12	12.5	26	27.1	18	18.7	7	7.3	0	0.0
48	24	25.0	14	14.6	7	7.3	4	4.2	1	1.0
Total	155		525		776		462		74	

## Data Availability

The excel file with the primary data can be obtained from the corresponding authors.
